# Not all benefits are equal: Incentives to seek reward and avoid penalty improve sustained attention in continuous performance tasks

**DOI:** 10.3758/s13414-026-03308-6

**Published:** 2026-07-16

**Authors:** Matthieu Chidharom, Edward K. Vogel, Monica D. Rosenberg

**Affiliations:** 1 Institute for Mind and Biology, Department of Psychology, The University of Chicago, 940 East 57th Street, Chicago, IL 60637 USA; 2 Department of Psychology, University of Chicago, Chicago, IL USA; 3 Neuroscience Institute, University of Chicago, Chicago, IL USA

**Keywords:** go/no-go, goal-competition, Attentional lapses

## Abstract

**Abstract:**

Sustained attention is notoriously difficult to maintain over time, with attentional lapses emerging rapidly during prolonged tasks. Motivation has been identified as a key factor in reducing these lapses and enhancing task engagement. According to recent accounts such as the goal-competition hypothesis, the perceived benefit associated with a goal helps sustain its active representation in working memory. These benefits can arise from the prospect of gaining a reward, avoiding a penalty, or both. However, it remains unclear whether avoiding a penalty – or the combination of penalty and reward – improved sustained goal maintenance to the same extent as pursuing a reward alone. To address this question, we recruited 30 participants to complete a “continuous performance task” (CPT) in which the type of benefit associated with the goal varied every 20 trials (reward, penalty, both, or no benefit). Replicating prior findings, we observed that rewards reduced attentional lapses compared to a no-benefit baseline. Avoiding penalties and combining both incentives also improved sustained attention in CPT, though the penalty condition was less effective than the combined condition. This suggests that while any perceived benefit can support goal maintenance, the motivational mechanisms may differ depending on the type of benefit – particularly when avoiding penalties alone.

**Open Practices Statement:**

The data and codes are available on the Open Science Framework repository at: https://osf.io/scmht/?view_only=df4ed739b7df40b19cc1ba00097450c5. It includes trial-level data, processed subject-level data, experiment task code, and analysis code. The study was not preregistered.

## Introduction

Sustained attention refers to the ability to maintain focus over time. However, this ability is difficult to preserve, and attentional errors or lapses in sustained attention occur frequently. A number of studies have investigated how to prevent such lapses by identifying factors that help improve sustained attention performance. Among these factors, motivation has emerged as a powerful contributor to maintaining task focus and resisting internal distractions. Although previous research has primarily focused on the effects of reward, few studies have examined the impact of penalties or the combined effect of rewards and penalties on improving sustained attention performance.

Researchers often investigate sustained attention using paradigms such as go/no-go tasks, traditionally referred to as “continuous performance tasks” (CPTs). In these tasks, participants are asked to press a button in response to frequent go stimuli and to withhold their response when infrequent no-go stimuli are presented (Robertson et al., 1997). Failures in sustained attention are typically measured by commission errors, defined as inappropriate responses on no-go trials. Two primary theoretical models have been proposed to explain when and why lapses of attention occur. According to overload theories, sustained attention depends on finite cognitive resources that gradually deplete over time, leading to higher error rates and disengagement (Baumeister et al., 1998; Smit et al., 2004). Alternatively, underload theories argue that lapses emerge due to boredom and insufficient cognitive stimulation, resulting in a more automatic and less attentive mode of responding (Manly et al., 1999; Robertson et al., 1997). Although both perspectives capture important facets of attentional decline, neither fully accounts for the range of empirical findings. For instance, if attentional failures were purely the result of cognitive exhaustion, motivational factors such as rewards should have minimal impact on performance. However, studies have consistently shown that rewards can enhance sustained attention (Esterman et al., 2014, 2016, 2017; Garner et al., 2024; Massar et al., 2016; Robison & Nguyen, 2023; Seli et al., 2015; Unsworth et al., 2025). On the other hand, if boredom were the main driver of lapses, increasing task difficulty should reduce attentional failures, yet heightened task demands often lead to more frequent lapses (Helton & Russell, 2011, 2013; Helton & Warm, 2008; Smit et al., 2004).

While traditional theories do not fully account for the diversity of empirical findings, new ideas are emerging to reconcile these results. One recent perspective suggests that lapses occur due to stronger competition between goals. In a philosophical article, Shepherd (2019) proposed an explanation for mind-wandering, a major source of lapses during CPT tasks (Andrillon et al., 2021; Bastian & Sackur, 2013; Chidharom et al., 2025a; Chidharom & Bonnefond, 2023; Kane & McVay, 2012; McVay & Kane, 2009). He argued that mind-wandering happens when the mind seeks to engage in tasks perceived as more beneficial than the current goal. This idea is particularly supported by research showing that the medial prefrontal cortex (mPFC) plays a key role in detecting and promoting beneficial or rewarded goals (Shenhav et al., 2013). For example, mPFC activity increases during periods of higher rewards both in humans (Kouneiher et al., 2009) and in macaques (Kennerley et al., 2011). In a similar manner, the mPFC is also active during episodes of mind-wandering, through its involvement in the default mode network (DMN), supporting the hypothesis that the brain may actively search for more rewarding tasks. This leads to an important idea: during sustained attention tasks, the current task goal is not simply held in focus by cognitive control, but remains in active competition with other goals that are momentarily less activated. As Oberauer (2024) highlights, individuals constantly maintain multiple goals and interests, even if only one is prioritized at a given moment (Oberauer, 2024).

Building on this idea, Chidharom et al. (2025b) proposed the goal competition hypothesis to explain the occurrence of attentional lapses. This hypothesis suggests that lapses in sustained attention occur when alternative goals compete with the primary goal and capture attention based on their perceived benefits. To test this hypothesis, the authors engaged participants in a switch-CPT, a sustained attention task in which participants had to alternate between two goals: categorizing faces or categorizing scenes. They found that during switch periods – when goal competition was higher – lapses in sustained attention occurred more frequently compared to repeat periods, when goal competition was lower. In a second experiment, participants were monetarily rewarded in one task but not the other, under the assumption that a higher perceived benefit would strengthen the activation of the rewarded task goal in working memory. The results showed a reduction in lapses for the rewarded goal compared to the unrewarded goal, suggesting that reward increases the sustained maintenance of the goal in working memory. Furthermore, switching to an unrewarded goal led to more lapses, suggesting that goals associated with greater perceived benefits are more distracting because they are more strongly competing with the current goal.

This goal-competition hypothesis of sustained attention fits well with previous theories of effort and fatigue. Kurzban et al. (2013) proposed the opportunity cost model, according to which the subjective experience of effort reflects the increasing value of alternative goals competing with the task at hand. From this perspective, mind-wandering can emerge as a rational reallocation of cognitive resources toward goals with higher expected utility. Similarly, Boksem and Tops (2008) cost–benefit model of mental fatigue conceptualizes fatigue as a motivational signal indicating diminishing returns from sustained goal pursuit. Within this framework, fatigue promotes disengagement from the current task when continued effort is no longer advantageous, a process that naturally aligns with a goal-competition account.

According to the goal-competition hypothesis, the activation of goals in working memory depends on their perceived benefits. These benefits can take different forms. The most classical and well-studied form is the expectation of a reward. When pursuing a goal is associated with the possibility of gaining a reward, it tends to enhance attention and reduce attentional lapses (Esterman et al., 2014, 2016; Massar et al., 2016; Seli et al., 2015). However, benefits may also stem from the prospect of avoiding a negative outcome or penalty. Despite this distinction, no study on sustained attention has investigated whether the avoidance of penalties can activate goal representations in working memory as effectively as the anticipation of rewards. Furthermore, it remains unknown whether the combination of both incentives – seeking rewards while avoiding penalties – can further enhance attentional stability. However, in everyday life, most goals are shaped by both motivational forces: the desire to gain something and the need to avoid failure or loss.

The aim of this study was to examine whether goals associated with avoiding a penalty or with a combination of reward and penalty help sustain task activation in working memory as effectively as reward-based goals. To test this, participants performed a CPT in which visual cues signaled the type of benefit associated with correct performance, with the benefit condition changing every 20 trials. In the reward condition, participants could earn a reward for each correct response on no-go trials. In the penalty condition, errors on no-go trials resulted in a loss. In the combined condition, both reward for correct performance and penalty for errors were in effect. In the neutral condition, performance had no associated consequences. If avoiding penalties or combining rewards and penalties supports goal activation in working memory similarly to pursuing rewards, we hypothesized that attentional lapses would be reduced in all benefit-related conditions compared to the neutral condition. Likewise, reaction time variability – an index of attentional stability – was expected to decrease during periods in which performance carried a motivational benefit.

## Method

### Participants

Thirty subjects participated in this in-person study (mean age = 24.8, SD = 3.8 years; 16 females, 14 males). An a priori power analysis was conducted using G*Power 3.1 to determine the required sample size. In a similar task, the effect of reward on no-go errors was large (f =.86) (Chidharom et al., 2025b). The analysis revealed that a minimum of five participants would be needed to achieve 95% statistical power. This estimation was based on a design including one group and four repeated measurements, assuming a correlation of 0.5 among repeated measures and a nonsphericity correction of 1. To allow for parametric statistical testing, 30 participants were included. All participants reported normal or corrected-to-normal vision and normal color perception. Exclusion criteria included any history of neurological disorder. All participants provided informed consent, and the study protocol was approved by the local ethics committee. Subjects were compensated for their participation.

### Stimuli

The stimuli consisted of 64 images of numbers and letters. The numbers included even digits (2, 4, 6, 8) or odd digits (1, 3, 5, 7) and the letters included vowels (A, E, I, U) or consonants (B, C, D, F). Each letter and number image was presented in four different fonts to create variability. These categories served as go or no-go stimuli or as distractors. For example, if participants were engaged in the letter task, they were instructed to press for vowels (go) but withhold their response for consonants (no-go) and numbers served as distractor stimuli. Indeed, on each trial, one number image and one letter image appeared simultaneously, one positioned to the left and one to the right of a central fixation point. Each image measured approximately 9 × 9 cm and the two images were spaced about 10 cm apart center-to-center. At the beginning of each block, a cue was presented to the participant to indicate the reward and cost condition. This cue consisted of two squares measuring 3 × 3 cm, positioned above and below the fixation point and spaced approximately 2 cm apart center-to-center.

### Procedure

**Fig. 1:**
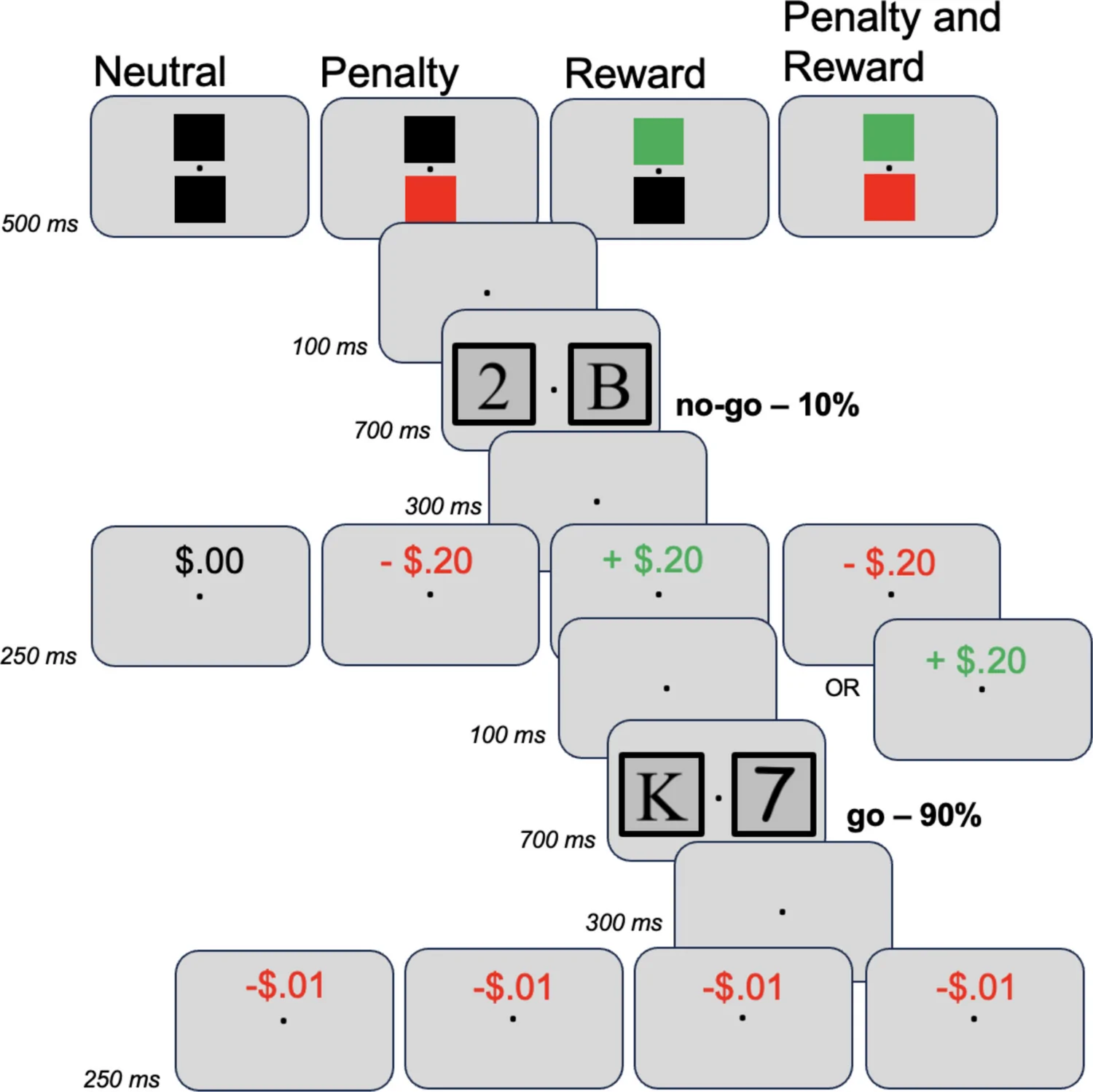
Sequence of the bilateral “continuous performance task” (CPT) – an example of the number task. In this example, participants are instructed to focus on numbers and press the response key as quickly as possible for odd numbers (go trials, 90%) and to withhold their response for even numbers (no-go trials, 10%). Each block begins with a cue indicating the motivational condition. Two black squares signal a neutral condition with no reward or penalty for no-go trials. A black and red square indicates a penalty condition, where a 20-cent deduction is applied for each no-go error (i.e., attentional lapse). A black and green square indicates a reward condition, where a 20-cent reward is given for each correct inhibition on no-go trials. Finally, red and green squares indicate a combined condition, in which both rewards and penalties are applied. In all block types, omission errors on go trials were penalized by –1 cent to ensure participants remained engaged and responded on go trials. Each mini-block lasts for 20 trials

Participants were engaged in either the letter task or the number task, counterbalanced across participants (Fig. 1). In the letter task, go trials (90%) were consonants and no-go trials (10%) were vowels, or vice versa depending on the counterbalancing across participants. In the number task, go trials were either even or odd numbers, and no-go trials were the opposite category, again depending on the counterbalancing. Each block began with a 500-ms cue indicating the type of motivational value associated with upcoming performance. In blocks with no associated value (neutral), two black squares were displayed. In blocks involving a potential penalty, the lower square appeared in red. In blocks offering a reward, the upper square appeared in green. In blocks combining both incentives, the cue displayed both a green upper square and a red lower square.

After the cue, a fixation cross was shown for 100 ms, followed by the simultaneous presentation of two images for 700 ms – one relevant to the current task set (e.g., letters), and one from the irrelevant category (e.g., numbers), with stimulus side randomized across trials. Participants had up to 1,000 ms after the stimulus onset to respond by pressing the spacebar. Feedback was then provided for 250 ms based on accuracy and block condition. In reward blocks, correct inhibitions to no-go trials earned + $0.20, displayed in green. In penalty blocks, an incorrect no-go response led to a loss of -$0.20, displayed in red. In blocks combining both, participants either gained or lost money based on their no-go performance. In blocks with no value, feedback to no-go trials simply showed $0.00 in black. To maintain motivation for go responses, omission errors on go trials always resulted in a -$0.01 penalty, displayed in red. The participant could earn up to an additional $10 extra based on performance, on top of the $20 base payment.

The task consisted of a single block lasting approximately 35 min. This block was composed of 56 mini-blocks of 20 trials each (1,120 trials in total), during which participants alternated between the four conditions (Neutral, Penalty, Reward, and Combined). The four conditions were counterbalanced randomly within the block. Participants first completed a short practice block of 20 trials with 50% of no-go trials to familiarize themselves with the task. Participants were required to verbally explain the meaning of the cue colors in the task and to achieve 90% accuracy in the practice block before performing the main task.

### Data and statistical analysis

Classical indicators of CPT performance were measured. The percentage of commission errors was calculated as the number of errors on no-go trials divided by the total number of no-go trials. Mean reaction time (RT) was computed based on correct responses on go trials. The coefficient of variation was calculated by dividing the standard deviation of RTs by the mean RT for each condition. To assess the effect of motivational value on performance, a repeated-measures ANOVA was conducted with incentive type as a within-subject factor including four levels: no value (neutral), reward, penalty, and combined reward and penalty. When significant effects were observed, post hoc paired t-tests were used to identify differences between incentive types. Tukey’s correction was applied to account for multiple comparisons. Furthermore, to examine whether improvements in one incentive type were associated with improvements in others, Pearson correlations were computed on difference scores. These scores reflected the reduction in commission errors relative to the neutral condition – that is, the percentage of errors in the neutral condition minus the percentage of errors in the reward, penalty, or combined conditions. A two-tailed alpha level of.05 was used for all statistical tests.

An exploratory analysis was also conducted to examine how the different incentive types modulated attentional fluctuations. Periods of being “in the zone” and “out of the zone” were calculated based on RT variability, following the method described by Esterman et al. (2013). We measured how consistent each participant’s RTs were by calculating the variance time course (VTC). To standardize the data, each participant’s RTs were first normalized, allowing comparisons on the same scale. The VTC was based only on correct responses during go trials, with each trial’s score reflecting how much that RT differed from the participant’s average RT. For trials with no responses or errors, such as missed go trials or correctly withheld no-go trials, RT values were estimated using linear interpolation from surrounding trials. We then split performance into two categories using the median VTC value across blocks: “in-the-zone” (low RT variability) and “out-of-the-zone” (high RT variability).

## Results

### Commission errors

**Fig. 2:**
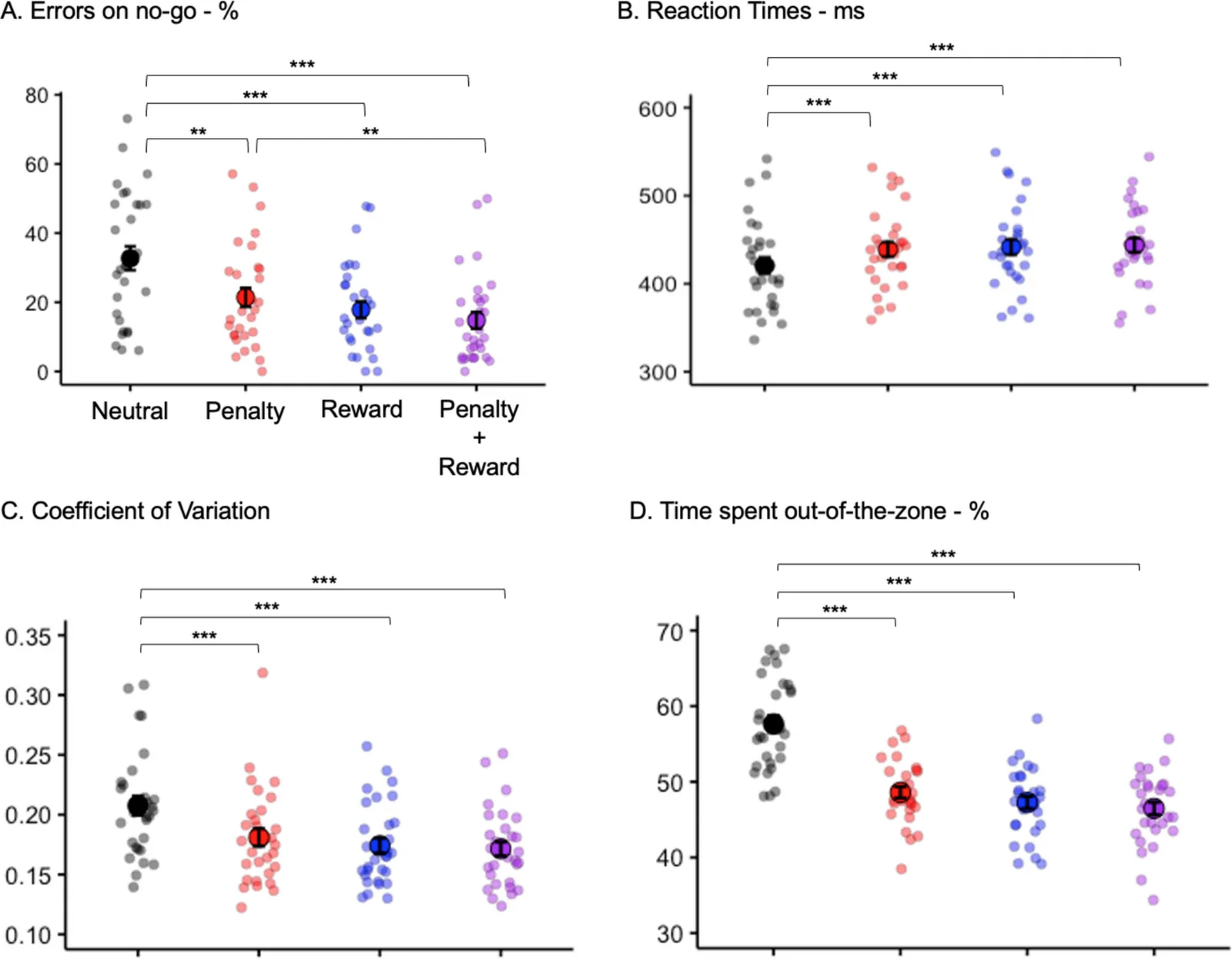
Behavioral results as a function of motivational condition. (**A**) No-go errors were reduced in the motivational conditions compared to the neutral condition. No-go errors were further reduced in blocks combining penalty and reward compared to the penalty-only condition. Participants were slower (**B**), less variable (coefficient of variation, CV) **(C)**, and spent less time out-of-the-zone (**D**) in the motivated blocks compared to the neutral ones. **p <.01; *p <.001. Error bars represent the standard error of the group mean

The ANOVA performed on no-go errors revealed a significant main effect of incentive type, *F*(3,87) = 19.80,* p* <.001, η^2^ₚ =.406. Post hoc comparisons showed that participants made significantly fewer no-go errors when performance was associated with both reward and penalty (*M* = 14.8%, *SE* = 2.36), *t*(29) = 5.42,* p* <.001, *p*_tukey_ <.001, *d* =.99; with reward only (*M* = 17.8%, *SE* = 2.41), *t*(29) = 5.27,* p* <.001, *p*_tukey_ <.001, *d* =.96; and with penalty only (*M* = 21.4%, *SE* = 2.73), *t*(29) = 3.85,* p* <.001, *p*_tukey_ =.003, *d* =.70; compared to the no-value (neutral) condition (*M* = 32.7%, *SE* = 3.45). Moreover, participants made significantly fewer errors when both reward and penalty were combined than when only a penalty was at stake, *t*(29) = 3.68,* p* <.001, *p*_tukey_ =.005, *d* =.67. No other comparisons reached significance (all *p*_tukey_ >.237) (Fig. 2A).

### Reaction times

The ANOVA performed on RTs revealed a significant main effect of incentive type, *F*(3,87) = 22.40,* p* <.001, η^2^ₚ =.436. Post hoc comparisons showed that RTs were significantly slower when participants expected a penalty (*M* = 439 ms, *SE* = 8.19), *t*(29) = 4.963,* p* <.001, *p*_tukey_ <.001, *d* =.91; when they expected a reward (*M* = 442 ms, *SE* = 8.68), t(29) = 6.019,* p* <.001, *p*_tukey_ <.001, *d* =.1.09; and when both reward and penalty were anticipated (*M* = 444 ms, *SE* = 7.97), *t*(29) = 5.778,* p* <.001, *p*_tukey_ <.001, *d* = 1.05; compared to the no-value (neutral) condition (*M* = 420 ms, *SE* = 9.43). No other pairwise comparisons reached significance (all pₜᵤₖₑᵧ >.160) (Fig. 2B).

### Coefficient of variation

The ANOVA performed on the coefficient of variation (CV) revealed a significant main effect of incentive type, *F*(3,87) = 20.40,* p* <.001, η^2^ₚ =.413. Post hoc comparisons revealed significantly reduced RT variability when a penalty was at stake (*M* = 0.181, *SE* = 0.00733), *t*(29) = 4.389,* p* <.001, *p*_tukey_ <.001, *d* =.80; when a reward was available (*M* = 0.174, *SE* = 0.00624), *t*(29) = 5.653,* p* <.001, *p*_tukey_ <.001, *d* = 1.03; and when both reward and penalty were combined (*M* = 0.171, *SE* = 0.00589), *t*(29) = 5.814,* p* <.001, *p*_tukey_ <.001, *d* = 1.06; compared to the neutral condition (*M* = 0.208, *SE* = 0.00800). No other comparisons reached significance (all *p*_tukey_ >.122) (Fig. 2C).

### Individual differences in motivational benefits

**Fig. 3:**
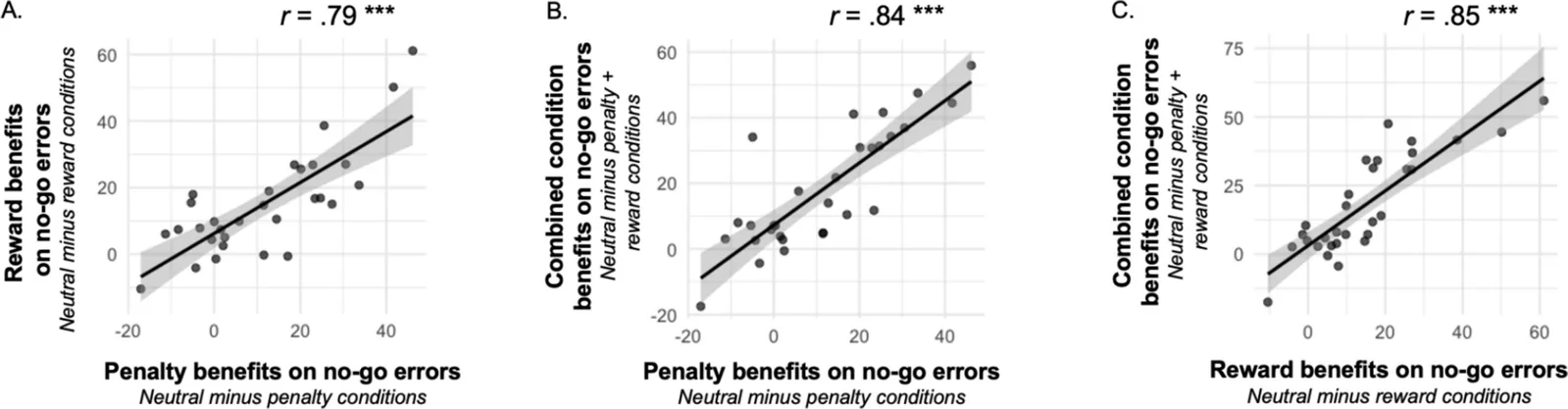
Pearson correlations between the benefits induced by different types of incentives on attentional lapses. Individuals who showed reductions in no-go errors in the penalty condition also benefited from the reward condition (**A**) and the combined condition (**B**). Likewise, individuals who benefited from the reward condition also benefited from the combined condition (**C**). *** p <.001

To explore whether individuals who reduced their attentional lapses when avoiding a penalty were the same as those who benefited from reward or from a combination of reward and penalty, Pearson correlation analyses were conducted. Results revealed strong and significant positive correlations between reductions in lapses under the penalty condition (neutral – penalty) and those observed under the reward condition (neutral – reward), *r*(28) =.792,* p* <.001 (Fig. 3A), as well as under the combined condition, *r*(28) =.839,* p* <.001 (Fig. 3B). Similarly, reductions observed under the reward condition were strongly correlated with those under the combined condition, *r*(28) =.852,* p* <.001 (Fig. 3C). These results suggest that individuals who benefited from one type of motivational value also tended to benefit from the others. However, these findings should be interpreted with caution, as our complementary analyses indicated that the difference scores in accuracy showed limited reliability. Specifically, we conducted a random split-half reliability analysis on the difference scores using 1,000 random iterations. This analysis revealed modest reliability for the Neutral–Reward contrast (Spearman–Brown corrected *r* = 0.51, 95% CI [0.18, 0.73]) and the Neutral–Penalty contrast (*r* = 0.51, 95% CI [0.20, 0.73]), as well as higher reliability for the Neutral–Hybrid contrast (mean *r* = 0.66, 95% CI [0.44, 0.82]).

A partial correlation analysis was conducted to account for the possibility that the observed effects were driven by neutral performance – that is, participants with higher error rates in the neutral condition may have had more room for improvement. After controlling for the percentage of commission errors in the neutral condition, the correlations between performance improvements across motivational conditions remained significant. Specifically, reductions in lapses under the penalty condition remained significantly correlated with those under the reward condition, *r* =.617,* p* <.001, and under the combined condition, *r* =.702,* p* <.001. Similarly, reductions under the reward condition remained correlated with those under the combined condition, *r* =.675,* p* <.001. These findings indicate that the observed relationships are not solely driven by differences in neutral performance.

### Exploratory analysis: Time spent out-of-the-zone

Attention fluctuates between periods of high and low attentional states. This analysis aimed to examine to what extent the incentive types reduce the frequency of out-of-the-zone periods, associated with poorer attention. As a preliminary step, we verified that in-the-zone and out-of-the-zone periods did indeed reflect fluctuations in performance. A paired-samples *t*-test revealed that no-go errors were significantly higher during out-of-the-zone periods (*M* = 25.1%, *SE* = 2.92) compared to in-the-zone periods (*M* = 19.1%, *SE* = 2.11), *t*(29) = –3.01,* p* =.005, *d* = –0.550. In a second step, we tested whether the percentage of time spent out-of-the-zone was reduced by the presence of motivational incentives. The ANOVA performed on the percentage of out-of-the-zone periods revealed a significant main effect of incentive type, *F*(3,87) = 25.60,* p* <.001, η^2^ₚ =.469. Post hoc comparisons showed significantly fewer out-of-the-zone periods when participants anticipated a penalty (*M* = 48.6%, *SE* = 0.745), *t*(29) = 5.587,* p* <.001, *p*_tukey_ <.001, *d* = 1.02; when they expected a reward (*M* = 47.3%, *SE* = 0.811), *t*(29) = 6.525,* p* <.001, *p*_tukey_ <.001, *d* = 1.19; and when both reward and penalty were involved (*M* = 46.5%, *SE* = 0.842), *t*(29) = 6.591,* p* <.001, *p*_tukey_ <.001, *d* = 1.20, compared to the neutral condition with no associated motivational value (*M* = 57.7%, *SE* = 1.106). No other comparisons reached significance (all *p*_tukey_ >.265) (Fig. 2D).

## Discussion

**Fig. 4:**
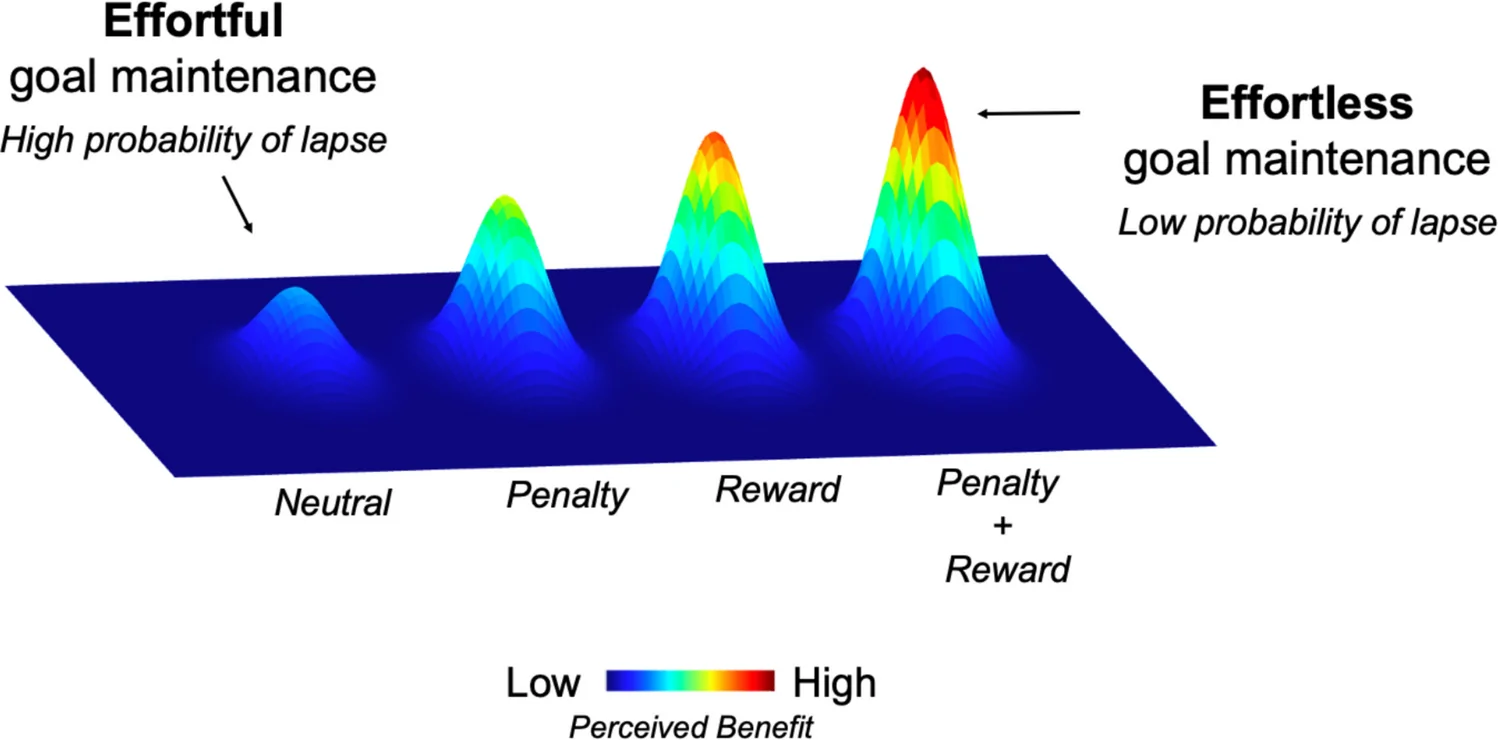
Representation of goal activation in working memory as a function of participant motivation. According to the goal-competition hypothesis, goal activation in working memory is influenced not only by cognitive control but also by the level of associated benefits. A goal with little or no benefit (neutral) will be weakly activated in working memory compared to conditions involving incentives (penalty avoidance, reward seeking, or the combination of both). It can be assumed that the combination of reward and penalty leads to stronger goal activation than penalty alone, potentially explaining the higher number of no-go errors observed in the penalty-only condition

The goal of this study was to examine the extent to which avoiding a penalty or combining a penalty with a reward can reduce lapses of attention in CPT. Our results show that, compared to a neutral condition with no motivational value, goals involving either penalty avoidance or a combination of reward and penalty significantly reduced attentional lapses, similarly to reward. Interestingly, the combination of both incentives led to greater improvements in sustained attention than penalty avoidance alone, suggesting a cumulative effect of the two types of motivational benefits. Furthermore, individuals who benefited from one type of motivational incentive also tended to benefit from the others, suggesting a shared motivational flexibility effect across incentive types. Overall, these results suggest that the benefits associated with a goal – whether through reward pursuit, penalty avoidance, or their combination – enhance the sustained activation of that goal in working memory, thereby extending the role of benefits within the framework of the goal competition hypothesis (Fig. 4).

Our results show that pursuing a goal aimed at avoiding a penalty is just as effective for sustaining attention over time as pursuing a goal aimed at obtaining a reward. Indeed, no-go errors were similarly reduced in both the penalty and reward conditions compared to the neutral condition. These findings extend previous research showing that rewards reduce no-go errors in sustained attention tasks (Chidharom et al., 2025b; Esterman et al., 2014, 2016, 2017; Massar et al., 2016; Seli et al., 2015). From the perspective of goal competition, these findings reinforce the idea that benefits – including those arising from penalty avoidance – support the activation and sustained maintenance of task goals. Interestingly, other reward-related manipulations have been used in the literature to improve sustained attention performance, such as the use of points to gamify the task or temporal incentives that shorten the task duration as a function of performance (Garner et al., 2024; Robison & Nguyen, 2023; Unsworth et al., 2025). Whether a penalizing version of such manipulations (e.g., making the task longer following poor performance), as well as a combined incentive approach, would replicate the results of the present study remains an open question.

Regarding the effect of incentives on RTs, one can speculate that motivation increases overall the engagement of cognitive control during the performance of sustained attention tasks. Indeed, RTs were generally slower in incentive conditions compared to the neutral condition. This slowing down of RTs is well documented in sustained attention tasks as a mechanism for reducing lapse rate (DeBettencourt et al., 2019). For example, a recent study showed that slowing down after a no-go error is an adaptive control mechanism that allows one to reduce the likelihood of committing another error on the next no-go trial (Chidharom et al., 2025a). It can suggest that slow RTs can involve cognitive control to reduce lapses of attention (Chidharom et al., 2026). RT variability was also reduced under incentive conditions. Specifically, the coefficient of variation – which reflects the standard deviation of correct reaction times normalized by the mean – was significantly lower in all incentive conditions compared to the neutral condition. Reduced RT variability has been associated with stronger engagement of cognitive control at both the intra-individual level (Chidharom et al., 2021a, 2021b, 2021c; Cooper et al., 2015) and the inter-individual level (Chidharom et al., 2021a, 2021b, 2021c; Cooper et al., 2017). While this explanation aligns with the overload theory, which suggests that cognitive control is central to sustained attention performance, it does not fully account for all the results. For example, Esterman et al. (2013) showed that periods of high RT variability – so-called out-of-the-zone states – were paradoxically associated with increased engagement of cognitive control, creating a theoretical contradiction with earlier interpretations (see also Chidharom et al., 2025c). An alternative explanation, grounded in the goal competition framework, proposes that benefits enhance the initial activation of task goals in working memory (Fig. 4). As a result, fewer cognitive resources are required to select and maintain goals over time, compared to less motivating goals that demand more resources to remain active. When a goal is perceived as beneficial, this may free up cognitive resources for engaging other effective mechanisms, such as proactive control. Thus, reduced variability in RT may not reflect greater engagement of control per se, but rather a reduction in competition from alternative goals.

In line with these findings, our exploratory results show that out-of-the-zone periods were reduced during incentive conditions compared to the neutral condition, suggesting that motivation helps minimize attentional fluctuations toward low-attention states. This replicates previous findings by Esterman et al. (2016), who showed that in-the-zone periods increased during reward conditions. Our study further refines this observation by demonstrating that penalty-based incentives can also contribute to stabilizing attention. Again, out-of-the-zone periods may reflect moments of heightened competition from task-irrelevant goals. Associating benefits with the task goal may enhance its activation in working memory and reduce competition from alternative goals, which could help explain and characterize the emergence of in-the-zone periods through the lens of motivation.

Beyond intra-individual effects, our study also shows that individuals who benefit from one type of incentive tend to benefit from others as well. Indeed, our correlation analyses revealed that improvements in no-go error rates induced by the three forms of motivation (penalty, reward, and their combination) were strongly correlated with each other, and these correlations remained significant even after controlling for neutral performance. This suggests that individuals who benefit from one type of incentive are generally able to take advantage of others as well, and this was independent of participants’ overall level of sustained attention performance. These correlations may appear counterintuitive in light of Atkinson’s (1957) motivational framework, which posits a dissociation between hope for success and fear of failure. One possible explanation lies in the distinction between subjective questionnaire-based measures and objective behavioral indices. Discrepancies between these types of measures are not uncommon. For instance, in a recent study we assessed fluctuations in sustained attention using both subjective reports (mind-wandering probes) and objective behavioral indicators (RT variability). The two measures were not correlated, suggesting a dissociation between subjective and objective indices of sustained attention (Bertschi et al., 2025; Chidharom et al., 2025a). Gaining a better understanding of how individuals flexibly adjust their attention in response to motivational incentives – and why others fail to do so – may help shed light on sustained attention deficits observed in clinical populations such as attention-deficit hyperactivity disorder (ADHD) or depression, where motivational impairments are frequently reported (Grahek et al., 2019; Modesto-Lowe et al., 2013; Volkow et al., 2011). These interindividual differences results should nevertheless be replicated in a larger sample in order to address the low reliability of the difference scores.

To conclude, this study highlights that sustained attention, as measured by a CPT, can be enhanced not only by goals associated with rewards, but also by those aimed at avoiding penalties. However, penalty-based goals were not as effective as goals combining both reward and penalty, which appeared to be the most effective strategy for maintaining attention over time. These results enrich the goal-competition hypothesis by showing that a variety of benefits – from reward to penalty avoidance – can activate and sustain goals in working memory. Future studies will be needed to investigate the neural mechanisms underlying these effects, in order to better understand how motivation interacts with sustained attention in both healthy individuals and clinical populations with motivational deficits. The effects of different incentive types on time-on-task performance also need to be further characterized, as does the extent to which these findings generalize to other forms of sustained attention beyond CPT and go/no-go tasks.

## Data Availability

The data and materials are available on the Open Science Framework repository (https://osf.io/scmht/?view_only=df4ed739b7df40b19cc1ba00097450c5).

## Code availability

The codes are available on the Open Science Framework repository (https://osf.io/scmht/?view_only=df4ed739b7df40b19cc1ba00097450c5).
